# New lessons from biology for economics and business: a systems approach to non-market environments

**DOI:** 10.1098/rsif.2024.0457

**Published:** 2024-10-30

**Authors:** YunHee Lee, Colin Mayer, Dennis Snower, Denis Noble

**Affiliations:** ^1^ Macoll Consulting Group, and Department of Medicine, Graduate School of Konkuk University, Seoul, Republic of Korea; ^2^ Blavatnik School of Government and Said Business School, Oxford University, Oxford, UK; ^3^ Professorial Research Fellow, Institute for New Economic Thinking, Oxford University, Oxford, UK; ^4^ Department of Physiology, Anatomy & Genetics, Oxford University, Oxford, UK

**Keywords:** Systems Public Affairs, systems biology, non-market strategies, purpose, profits, delegation

## Abstract

Drawing on recent advances in biology, this paper describes a systems approach, ‘Systems Public Affairs’ (SPA), to integrate non-market strategies in corporate purposes and strategies. Just as the environment of organisms affects and is affected by their development and evolution, so individuals and businesses adjust to and can shape their non-market environment, which we define as ‘a historically formed national and social sphere, including laws, regulations, and policies, which supports, maintains and restrains the operation and preservation of markets’. The paper uses cases from South Korea to illustrate this approach. Emergent ideas in biology have great relevance for micro-foundations of business. Traditionally, economic activities are outcomes of individualistic decision-makers, each promoting their distinct objectives within economic markets. In the SPA approach, decision makers in the domains of business, policy and civil society collaborate in shaping non-market environments to align business objectives with public interest. This requires agency to rise to higher levels than that of businesses, policymakers and civil society through collaboration and experimentation in the presence of stochasticity and radical uncertainty. Analogous to the advancement of organism evolution through emergence of nervous systems and learning, so alignment of organizations with their non-market environments accelerates economic and social development.

## 1. Introduction

One of the most profound analogies between the natural and the social sciences is between gene-centric theories and natural selection in biology, and utility maximizing agents and market competition in economics. Both lead to the conclusion that the evolution of life and social well-being can be derived from the properties of organisms at the micro-level of genes in biology and individuals and businesses in economics.

Concepts and mathematical models in gene-centric biology and in micro-economics share many features. The free market (invisible hand) models use the same reduction to the micro-level as gene-centric evolutionary biology (‘reductionism’), and market selection of winners over losers resembles the natural selection of (blind) mutations.

However, there have been many developments in biology challenging gene-centric theories that have profound implications for our understanding of the functioning of businesses and economies. Systems biology of metabolic networks has already shown, 50 years ago, that ‘varying amounts of control can be distributed over the enzymes of the pathway, but this is a property of the metabolic system as a whole and cannot be predicted from the characteristics of the enzymes in isolation’ (citation from Wikipedia entry on Henrik Kacser concerning Kacser and Burns 1973: https://en.wikipedia.org/wiki/Henrik_Kacser.) ([1]; see also [2,3]) .

More recently, Duarte *et al.* [4] have demonstrated that models of metabolic networks reveal many ways in which metabolic integrity can be maintained. In yeast, 80% of gene knockouts are silent in normal physiological conditions [5], while cardiac rhythm was first shown to be robust against knockout or block of individual genes and proteins over 30 years ago [6].

Many more discoveries are now acknowledged as showing that ‘genes are not the blueprint for life’ [7,8, p. 254]. Among the most relevant to this paper are:

- evidence for epigenetic (non-DNA) acquired characteristics in response to environmental change [9–11], some of which are transmitted to the germline [12];
- evidence for the dependence of accurate replication of genomes on the physiologically controlled error-correcting processes in living systems [13–15], implying that DNA cannot any longer be claimed to be an accurate *self*-replicator; and
- evidence of the failure of polygenic scores to be practically useful in predicting many disease states [16].

Furthermore, recent work shows that the causal consequences of the principle of biological relativity, i.e. no privileged level of causation, apply only between the physical levels of biological organization [17,18], whereas social forms of organization may have priority [19,20], at least in the case of human biology. The interaction between the social and physical forms should be viewed as an alignment, rather than as physical causation to which the principle of biological relativity necessarily applies. That necessity arises from the nature of differential equation models: higher levels of physical organizations form the boundary conditions for the lower levels. Immaterial causes clearly cannot perform this function.

In the economic world, top-down causation takes the form of higher level agency, whereby micro-level actors are induced to collaborate in such a way that decision-making effectively lies at the group level. The implications of this for analogies between biology and the social sciences are profound, since top-down causation in the material and immaterial worlds both no longer support a reductionist perspective in the social sciences.^1^

In particular, the recognition of the limitations of gene-centric theories and the importance of epigenetic factors as explanations of the characteristics of organisms is paralleled by a similar appreciation of the significance of non-market factors in business and economics, in addition to market factors. Just as the nervous system established systems of learning that accelerated biological evolution, so too organizational cultures and values permit them to develop explanatory and predictive mechanisms for accelerating their evolution in non-market environments.

In this paper, we introduce the concept of Systems Public Affairs (SPA) to take account of this fundamental shift in viewpoint in biology to economics and the social sciences. SPA is a management strategy to promote the sustainability of businesses in responding to growing complexity and uncertainty in non-market environments. It sheds light on the importance of non-market strategies in assisting businesses with managing uncertainty and complexity in line with their purposes, profitability and continuity, and governments with promoting social and environmental flourishing, prosperity and sustainability. It is critical to creating a framework that integrates the currently fragmentary non-market strategies employed by companies in a systems approach at the early stages of corporate purpose formulation and strategy planning.

The relevance of biology to current economic and management models was described in Lee *et al.* [22], which used analogies with multi-level causation and symbiogenetic relationships in biology to show that cooperative strategies can be more beneficial to corporations. That analysis was underpinned by the principle of biological relativity [23].

This article extends that analogy by incorporating recent findings of social causes having priority in organism behaviour [18,19]. The corresponding problem in economics is the failure of traditional micro-economic models to anticipate and shape the social, political and environmental contexts in which businesses operate and need to address. Learning and understanding of the influence of non-market environments may accelerate evolutionary processes that rely on traditional natural selection processes associated with markets.

Traditional selection processes associated with market competition can be accelerated and aligned with the public interest through two major channels: (i) adjustments of the internalized objectives of market participants through the evolution of social norms and values, and (ii) niche construction through the development of institutions and organizations that reward benefiting and sanction harming the public interest. In the latter context, much government policy may be usefully understood as a process of artificial selection of top-down decision-making processes, influencing the degree to which businesses collaborate with one another and with other public- and private-sector decision-makers to pursue the public interest. Case studies are included to demonstrate the success of the SPA approach in real-life scenarios.

Section 2 describes non-market strategies and SPA. Section 3 discusses advances in our understanding of biological boundary conditions and their relevance for adopting SPA in business. Section 4 summarizes three cases of the application of SPA. Section 5 concludes the article.

## 2. Non-market strategy and Systems Public Affairs

### 2.1. The importance of non-market strategy

We define the non-market environment for corporate activities as ‘a historically formed social and national sphere, including laws, systems, regulations, and policies which promotes, maintains and restrains the operation and preservation of markets’. Instead of regarding the non-market environment as a naturally formed sphere separate from the market, it is viewed as an institutional sphere which promotes the functioning of the market. There is a social nesting hierarchy comparable to that of levels of organization in biology.

Typically, a market is regarded as a system in which economic transactions between businesses, suppliers and consumers are made and prices set according to certain property right principles, contracts and standards [24]. In non-market environments, transactions between businesses, individuals, stakeholders, governments and the public are structured by social, political and legal arrangements and institutions [25]. The non-market environment includes all the social and political relationships and contexts in which businesses operate [26,27].^2^

The subject of management studies recognizes the importance of the business environment and describes its influence on business strategies that reflect information on markets and industries [26,30]. There are significant differences between business engagement in the market and non-market domains. First, whereas a business can compete with other businesses in pursuit of profit, it generally requires cooperation with other entities in the non-market domain. Second, while businesses must respond flexibly and swiftly to changes in consumption patterns in the market, non-market relations require long-term relationships built on trust and goodwill. Finally, while profit is the primary objective in the market, the non-market environment is driven by a variety of social norms and values. As a result, as in biological models of cooperation [7,31], for businesses to manage non-market strategies efficiently, adaptation and acceptance is more important than competition, and cooperation in a non-market environment is essential.

A systems approach to non-market strategy emphasizes the need for business to promote the non-market environment through SPA.^3^ For business to play a socially and environmentally responsible role in socio-economic systems, it is important that businesses manage their non-market environment not just with their own profit objectives in mind, but rather with a view to creating a non-market environment within which business’s pursuit of profit becomes compatible with the public interest. Such creation is in fact co-creation since businesses cannot fully achieve the requisite transformation on their own. The non-market environment of business needs to be negotiated by policymakers, business leaders and civil society representatives. The aim of the negotiations is the creation of a business setting that aligns private and public objectives by permitting business leaders to compete in pursuit of profit while simultaneously benefiting society and the environment. In a business setting in which such alignment occurs, decision-making power is no longer located independently at the levels of policymakers, business leaders and civil society representatives, but rises to a higher level that encompasses the public interest.

Such a non-market environment of business requires a redefinition of profit that recognizes the complementarity between market and non-market considerations. Any business activity that benefits society and the environment should be measured as a generator of profit, whereas any activity that harms society and the environment should be measured as a diminution of profit. In short, social and environmental problem solving should be the source of corporate profit, and social and environmental problem-creation should incur costs of remedying and rectifying detriments that diminish profit [33–35]. This redefinition should be the basis for business reporting and accounting.

This goal can only be achieved when businesses join policymakers and civil society leaders in constructing a common understanding of the public interest, so that the legal system ensures that businesses take account, not only of their retained and distributed profits, but also the producer surplus, consumer surplus and employee surplus of business activities, the social contribution of corporate tax, stakeholder externalities (uncompensated net costs to firms’ stakeholders) and third-party externalities (uncompensated net costs to agents who are not among the firms’ stakeholders, e.g. unborn generations). In short, it involves a legal framework arising from cooperation between business, policymaking and civil society in the broad public interest [36].

In this context, SPA is the management of the non-market environment by business, government and civil society to promote an alignment between private incentives in profit with social interests in human and natural world flourishing. The non-market environment thereby ensures that businesses which create problems will no longer be able to gain a competitive advantage over their socially responsible counterparts. It therefore promotes markets by aligning private incentives with social and environmental interests and avoids unfair competitive advantage being gained at the expense of others.

### 2.2. Public affairs as a non-market strategy

Traditionally public affairs are discussed in the context of managing external relations through lobbying parliaments, governments, local communities and the media. This approach to public affairs is inadequate in the context of the non-market environment [37]. Instead, public affairs should be elevated from being a mechanism of handling external business relations to a strategic tool for managing the non-market environment in the public interest. As Hamel argues the most important question for business in the twenty-first century is, ‘can we change as fast as the world does?’ [38, p. 55]. The visible hand has as much, if not more, impact on business activities as the invisible hand.

Public affairs can be defined as ‘the management of the non-market environment to enable business to conduct its economic activities in an effective, pro-social and sustainable manner’. Public affairs in this context involves a comprehensive process of preparing, establishing, executing and evaluating a non-market strategy. It is a complement to a market strategy in the formulation of corporate strategy.

This approach to public affairs is dictated by significant changes in the non-market environment affecting relations between businesses, markets, society and nations. As we describe below, internationalization, national regulation, politicization of society, advances in information and communication technology (ICT) and increased corporate responsibilities are at the heart of these changes.

#### 2.2.1. Internationalization

The increasingly international and global nature of business since World War II has prompted the introduction of a large array of international agreements and standards to tackle such issues as the environment, fair trade, finance, taxation, copyright, bribery, corruption, modern slavery and human rights. These apply to businesses wherever they operate.

Nevertheless, over the past decade companies have repeatedly been found to be in violation of these standards. For example, Samsung Electronics’ suppliers have been accused of using conflict minerals from 62 refineries in 28 countries, Nike of harsh labour conditions in their manufacturing facilities, Citi Group of funding development projects that have destroyed tropical rain forests and Nestle of using cocoa from farms that employ child labour. In response, these companies now publish reports [39–42], which demonstrate how they are seeking to abide by not only international agreements but also globally acceptable standards of conduct.

#### 2.2.2. National regulation

In addition to international agreements, there has been a raft of new regulations introduced at national levels in response to privatization of utilities and public services around the world. Regulation constrains the discretion of business in how it runs its activities, and regulatory changes pose greater business risk for firms [43].

However, insofar as those regulations are designed to promote the common good, businesses need to find ways of ensuring that they succeed in fulfilling that objective. This requires a strong understanding on the part of business not only of formal legislation and regulatory rules but also the specific social norms, conventions and codes of conduct that that exist within and across countries in which they operate and lie behind them at local, regional, national and international levels.

#### 2.2.3. Politicization of society

Pressure on business comes not only from international agreements, national governments and regulators, but also from non-governmental organizations (NGOs), civil society and social media. A growing crisis of confidence and trust in businesses, governments and the public sector has encouraged the emergence of these groups as ways of rallying public opinion [25,43]. Energy and social media companies have particularly felt their force. These organizations play on the sensitivity of business towards consumer and public opinion. Greenpeace, Mighty Earth, Sum of Us and other private organizations have their own respective agenda, many growing beyond the boundaries of a single nation to gain international reach and impact.

Increasingly business is coming to appreciate the benefits of working in partnership instead of conflict with these organizations and use them as ways of anticipating future issues with which they might otherwise be confronted. McDonald’s, Coca-Cola and other businesses have conducted projects such as waste reduction, animal welfare, water replenishment and greenhouse gas reduction jointly with social movement organizations. For the purposes of our analysis, it is important that these initiatives are conducted not as public relations damage limitation exercises, but rather as positive alignments of their business purposes with the public interest.

#### 2.2.4. Advances in information and communication technology

The Internet and social networks are bringing about profound changes in how public opinion and policy are formed. Political issues no longer begin with national institutions determining public policies, but instead with grassroots movements using the Internet and social media to galvanize public opinion around public policy reform.

Such forms of public opinion formation and public policy pose real and unpredictable challenges for business. Companies are liable to be immediately exposed on the Internet and elsewhere to revelation of unethical or antisocial behaviour [38]. This has made the management of the non-market environment acutely important for business.

#### 2.2.5. Increased corporate responsibilities

On top of these developments, one that has most directly impacted on businesses is the rise of business social and legal responsibility. Society is increasingly demanding that businesses act as responsible global corporate citizens. By contrast to the traditional concept of businesses as a closed system, they should be seen as an open system taking responsibility for, as well as influenced by, its external environment.

At the heart of this is the question of the relationship businesses and societies should have with each other. The UN Global Compact has set global standards for responsibility in relation to, for example, environmental protection, sustainable development, human rights, child labour protection and anti-corruption [44], but even more is now happening at the national and regional level. There has never been a time when the depth, breadth and complexity of the interaction between business and the non-market environment have been more extensive.

What has prompted this is the emergence of multinational corporations and entrepreneurs with growing impact on environments and people’s lives. Globalization and automation have resulted in a decoupling of business interests from those of the communities in which business operates, as flexible global value chains have made business’s involvement with communities more transient and conditional on relative profitability. Enhanced corporate responsibility to society and the environment seeks to address this by aligning private and public incentives.

## 3. Biological boundary conditions and enacting Systems Public Affairs

SPA is a management strategy for businesses to take a more holistic view and manage their dynamics and boundary conditions in a world of increasing volatility, uncertainty, complexity and ambiguity. To implement SPA, there needs to be a new appreciation of the role of business in society and businesses must be equipped with internal organizational structures and capabilities in line with SPA that combine market and non-market environment strategies.

### 3.1. The biological boundary conditions

The non-market environment boundary conditions that underpin SPA may have their roots in analogies with the recent developments in biology described earlier in this article. Drawing analogies from biology is not new. The disciplines of economics, business and management have often interacted with and drawn conclusions from comparisons with previous models used in biology. Selfish-cooperative game theoretic models in economics sometimes use similar ideas and mathematics as those in selfish gene theory in biology. But that theory has now been shown to be an incorrect representation of living systems [45,46]. The social sciences can no longer draw on analogies with the biological foundations of selfish gene theory because simplistic gene-centric interpretations have been undermined by discoveries in molecular biology and physiology.

The technical details of these discoveries have been analysed in several recent articles and books [18,23,47,48]. The key conclusions relevant to the social sciences are as follows.

#### 3.1.1. One-way reductionist models are inadequate

**Figure 1.**
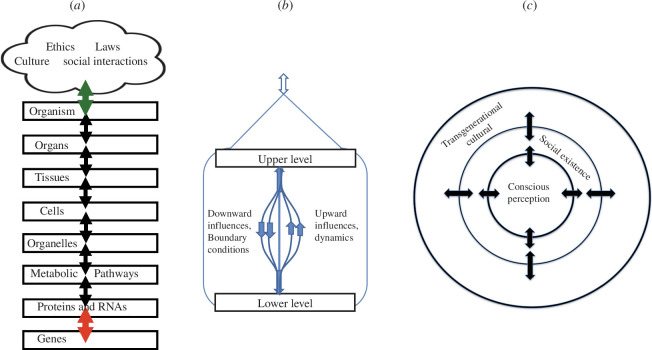
(*a*) Multi-level causation in multi-cellular organisms. The physical levels of organization are represented by labelled rectangles with double-headed arrows of causation between the levels. Non-physical causation is represented as a cloud to emphasize that social causation is immaterial. The double-headed arrow is coloured green to indicate that the forms of causation involved are not the same as between physical levels. Similarly, the bottom arrow between genes and proteins and RNAs is coloured red to indicate that a coding step is involved, a causation by form rather than mechanics. (*b*) Double-headed arrows are expanded to show that upward and downward forms of causation are instantaneous. This diagram applies between levels in which the upper level forms the boundary conditions constraining the lower level. ((*a*) and (*b*) are redrawn from [17], where further explanation can be found.) (*c*) Replacement of cloud with concentric circles to emphasize that social causation is consciously purposive and can generate transgenerational cultural evolution [31]. For further explanation on how immaterial and material causation may be represented as interacting without being subject to the principle of biological relativity, see Noble & Noble [18].

All physico-chemical processes in living systems are necessarily constrained by higher level organization [17]. This is true even for determinate physical processes. There is no causal closure without the initial and boundary conditions set by higher level constraints. Furthermore, the interaction between levels upwards and downwards is necessarily simultaneous since the upper level simply forms the boundary conditions for the lower level. It is not possible to separate out these multi-level processes in time (see figure 1*b*). This is an important feature of the principle of biological relativity. It does not apply to networks, such as a purely metabolic network, where all the processes are represented as occurring at one (in this case, molecular) level.

The distinction can be best understood by comparing a metabolic network, such as the citric acid (Krebs) cycle with the Hodgkin cycle. The Hodgkin cycle is necessarily multi-level: the membrane voltage changes constrain the molecular movements at the same time as those movements [49].^4^ We will draw the implications of this important fact for economics and management studies later in the article.

#### 3.1.2. Randomness is harnessed by living organisms

Standard evolutionary biology assumes that chance events cannot be used to produce purposive directional outcomes except through the process of natural selection involving the life or death of individual organisms. Yet, all organisms with immune systems clearly produce functionally directed outcomes by selecting among the random changes thrown up by chance mutations. We now know that this process of harnessing stochasticity occurs everywhere, not only in the immune system. The harnessing of stochasticity is general and is also the process by which organisms can be creative in the choices they make [18], including the values that underlie our social systems. Of course, non-living systems can also harness noise [51–54].

#### 3.1.3. Analogies with simplistic gene-centric biology are no longer a sound basis for social science theorizing

Lee *et al.* [22] drew extensively on the multi-level analysis of biology, using the principle of biological relativity [23]. That principle states that there are no privileged levels of physical causation within and between the levels of physical organization of living organisms. But the extrapolation to economics, business and management depends critically on organizations in which many of the levels of organization are not physical; they are necessarily levels of social organization.

Reinvestigation of the principle of biological relativity has shown that causation from social levels of organization cannot be subject to the same principle. There is a sense in which social factors are primary since they cannot be represented in mathematical models of physical processes where one level can form boundary conditions for a lower level. Organisms can however align their behaviour with social expectations [18,19]. But this does not mean that those social expectations dictate what organisms choose to do. The conclusion is rather that values matter, and they cannot be fully determined by the physical processes underlying the behaviour of organisms.

Figure 1*c* takes this analysis further, by representing the forms of social causation as concentric circles rather than as different physical levels of organization. The important conclusion of this is that the social sciences should no longer draw analogies from simplistic gene-centric biology. In relation to the determination of value and values, such as those that underlie SPA, those values must be sought in our culture and social networks. We humans should draw lessons from cell systems biology and physiology in exercising agency when deciding which structures of social interaction to choose to favour:

The psychosocial level is unique. If there is a privileged level of causation, then it lies at the psychosocial level and not at the level of genes. This is the level at which willful agency is initiated and organisms can be genuinely selfish or altruistic. In truth you cannot be selfish if you do not have the choice to be altruistic, which is why selfishness cannot be applied at a genetic level, neither metaphorically nor literally. [47, p. 69]

Such cooperation doesn’t involve an incident-by-incident ‘what’s in it for me’ assessment. Nor is it hard-wired or genetic. It is socially developed and culturally maintained by cooperation and social cohesion, not self-interest. In this sense the regulation constraint is not physical, it lies in the ideas about the world, which we may hold in common and develop with others. [47, p. 75]

There is therefore no sense in which these influences and decisions are determined by biology alone, other than that the existence of cognitive beings capable of making such choices depends on the biological processes involved. We cannot exist as human beings without the capacity to make proteins from our genome templates, but that capacity, in and of itself, does not determine what we choose as the social values underlying public affairs. Following the failure of the selfish gene theory in biology, it is important to replace its influence in economics and management with a different comparison, which, we argue, SPA can provide.

#### 3.1.4. Active agency is associated with later stages of evolution based on learning

In multi-cellular organisms, constraints on organisms depend on chemical communication from one part to another through, for example, hormones and transmitters. But chemical diffusion is a relatively slow process and action often needs to be faster. The electrical impulses conveyed through nervous systems provide the means for achieving that.

With a nervous system, the potential exists to harness not just stochasticity but also agency based on unlimited degrees of associative learning [55]. This extends the degree of intentionality in defining the purpose of organisms. In particular, the role of the environment in imposing constraints on lower level organisms morphs into a two-way communication channel in which the environment not only imposes constraints on the development of organisms, but organisms can also select between different random outcomes based on their conformity with the environment in which they operate.

In essence, there is a matching between the evolution of organisms and the nature of their environment, in which agency and consciousness allow for a degree of determinism in selection based on fulfilment of objectives and purposes. This substantially accelerates evolution by introducing intentionality and direction into otherwise slow natural selection among randomly determined outcomes.

**Figure 2.**
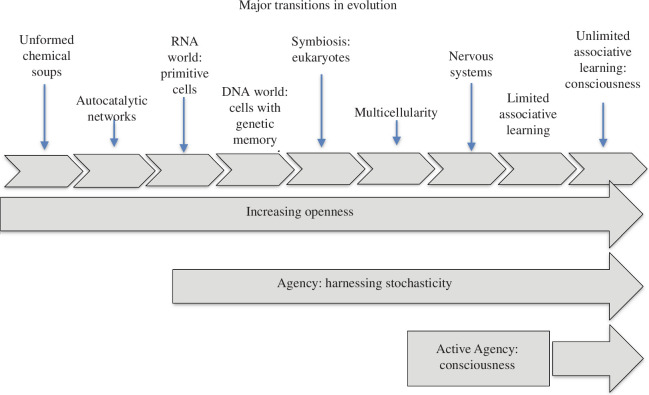
Distinguishes nine major transitions in the evolutionary process. Each transition facilitates later transitions, and organisms become increasingly open to interactions with other organisms, leading to forms of learning and anticipation involved in active (conscious) agency [47].

Note that these conclusions (figure 2) are significantly different from those drawn from the standard theory of evolution, i.e. the Modern Synthesis [56–58]. In particular:

- there is a clear directionality to the process since each transition generates the possibility of further new processes and transitions; and
- the diagram openly acknowledges the evolution of intentional agency, which is so central to our analogies between modern biology and the requirements of businesses in interaction with society.

#### 3.1.5. Implications for business, economics and the social sciences

These developments in biological knowledge and understanding have profound implications for the social sciences and especially business and economics. Table 1 below summarizes how (i) neo-Darwinian biology relates to shareholder primacy and competitive markets models of the firm, (ii) new biology differs from neo-Darwinian biology, (iii) SPA differs from shareholder primacy and competitive markets models of the firm, and (iv) new biology relates to SPA.

**Table 1.** Summary of comparisons between biology and business. First column: Neo-Darwinian biology. Second column: Business competition. Third column: New Biology. Fourth column: Systems Public Affairs.

neo-Darwinian biology	shareholder primacy and competitive markets	new biology	**Systems Public Affairs**
**gene-centric theory**	business follows shareholder interest only	phenotype-centric biology	society, culture and politics influences decision-making
**central dogma**	one-way flow from shareholder interest to decision-making	two-way flow between genes and phenotype: organisms can edit genes	flow is two-way involving both market and non-market strategies
**Weismann barrier:** **no soma to germline connection**	exclusion of non-market factors	inheritance of acquired characteristics via germline	inclusion of non-market factors
**DNA ‘replicates like a crystal’**	business boards self-perpetuate themselves	correct replication dependent on the cell	boards collaborate with policymakers and civil society in the public interest
**evolution is not purposive**	society values not relevant	organisms are purposive	evolution of culture and policymaking can be guided
**natural selection alone sufficient**	competition entirely sufficient	evolution speeded up by other processes, including social selection	policymaking can shape variation, selection and transmission of ideas in the public interest, so resulting in faster adaptation

The first implication is the role of business in responding to the stochasticity, i.e. the ‘radical uncertainty’ [59] to which economies are exposed. The characteristic of business that distinguishes it from many public sector organizations and institutions is its ability to experiment, learn and adapt to changing circumstances. Entrepreneurship, innovation and ‘creative destruction’ [60] are the hallmarks of successful businesses. Just as the evolution of organisms relies on stochasticity, so too innovative businesses harness and thrive on stochasticity to their advantage. Notions of evolution permeated economics with the work of Armen Alchian who argued that firms do not consciously strive to maximize profits but are effectively driven to do so by a combination of uncertainty, probabilistic outcomes and natural selection of the fittest [61].

Second, analogous to the significance of two-way flow in the new biology, so there is a growing realization of the environmental and social constraints within which businesses operate and the degree to which they grow, merge, divest and collapse. This was reflected in Richard Nelson and Sidney Winter’s seminal book on evolutionary economics [62], which developed Alchian’s work together with Richard Cyert and James March’s research on behavioural economics [63]. This views firms as operating within selection environments based on organizational routines they can evaluate and change.

Third, and this is where the paper differs from the existing literature, there is a greater consciousness and understanding of the environmental constraints that elicits a two-way interaction in which the non-market environment responds to the needs of business, and business learns how to adapt to its environmental constraints. Instead of taking the environmental constraints and selection mechanism as given, firms seek to modify them in such a way as to produce more profitable outcomes.

The interactions are two-way. As our figure 1 illustrates, in multi-level biology the interactions are necessarily simultaneous. The analogy for business is that interacting with non-market environments must be built-in to management systems and incentives, not simply imposed as external constraints. Failure to recognize this has resulted in the inability of regulation and ex post penalties to constrain companies from concealing detriments, lobbying against regulation, circumventing it and, if possible, turning it to their competitive advantage as a barrier to entry of new firms. It is only if law and regulation simultaneously ensure that ex ante, anticipated profits derive from social and environmental benefits not detriments that competitive markets are welfare enhancing.

That is the basis of SPA. It provides the means for accelerating the rate of evolution of business and economies. Whether that evolution is in a direction which promotes conformity between the objectives of business, societal and environmental depends on the motives that drive business. That is why the alignment between business pursuit of profits and problem solving, not creation for others, is such an essential prerequisite.

It is important to appreciate that this goes beyond notions of altruism or group as against individual selection (see for example [64], which defines altruism as ‘sacrifice of fitness’ (p. 158) and therefore ‘unlikely to survive, even if it enhances the fitness of the group as a whole – for while the whole group is growing, the altruists in it will gradually vanish, reducing and then eliminating the group’s initial advantage’ (p. 156). SPA often benefits from collaboration (that is cooperation beyond the bounds of enlightened self-interest) because adapting environmental constraints can be a public good. The provision of this public good does not require altruism, but rather works through mutualism and commensalism. Beyond that, adaptation of environmental constraints can be a private good where the benefits accrue to the firm that practices SPA and communicate to other firms through processes of learning, experimentation and adoption.

### 3.2. Enacting purpose in business for Systems Public Affairs

To carry out the redefined social role or purpose of business, businesses must communicate and cooperate with various stakeholders not just in the market, but in the non-market environment as well. Analogous to the inheritance of acquired characteristics in the new biology, the existing attitudes and ideas of corporate roles must embrace public affairs as a key component of corporate strategy. SPA is not something that is asked of business from outside but is a new management strategy in an era when the non-market environment is utilized for competitive advantage and value creation. Such a change in attitude positively impacts on the areas surrounding business.

First, businesses should not view the non-market environment as something that is given, or simply a set of costly regulations but an approach that is proactive and productive. Businesses can manage the non-market environment to grow their sphere of business beyond just transaction of goods and services. Second, businesses can enhance their adaptability to change by innovating and creating new markets and strategies. Third, businesses can facilitate social change by communicating and cooperating with other parties in the non-market environment. In other words, business can do more than just accommodate non-market environment changes after the fact, but utilize such changes to create economic value, competitive advantage and new markets, thereby facilitating social innovations.

Beyond this, businesses must choose to manage their non-market environment not just to maximize shareholder value, but to engage with policymakers and civil society representatives to co-create a legal and regulatory framework that will enable businesses to act in the global public interest in the processes of pursuing their own profit. Implementation of such non-market strategies carries visions of corrupt relationships between politics and businesses that put personal gain before public good.

This emphasizes the importance of corporate purpose. Businesses that see their objectives as being purely shareholder value and profit maximization will seek to influence the non-market environment in a way that is solely in the interests of themselves and their shareholders. However, businesses that adopt as their purposes promoting shareholder value through solving problems in the service of the public interest, not creating problems for others, align their own interests with the environment in which they operate.

The way to avoid conflict between corporate strategies with those of society is not therefore just to rely on regulation and public law to constrain the conduct of business but also to align the purpose and objectives of business with those of their non-market environment. This can be done through ensuring that corporate law embeds corporate purposes of producing profitable solutions for the non-market environment without profiting from producing problems at the heart of business. This process of contributing to the creation of socially enlightened law involves a new form of interaction between business and the legislative, executive and judicial branches of government. Beyond that, business can contribute to the evolution of social norms and governance systems to reinforce the business behaviour mandated by enlightened law.

This approach requires genuine collaboration between business, policymakers and civil society to design rules whereby businesses compete for profit while benefiting society and the environment. Such collaboration can be understood as the product of ‘guided evolution’ of economic, policy and social systems. Business, policymakers and civil society representatives need to (i) formulate clear targets of selection, determining which behaviour patterns are to be encouraged, (ii) determine the degree of variation permissible around these targets, i.e. the range of allowable experimentation, (iii) identify adaptive practices and their context dependence, and (iv) replicate these practices in analogous contexts. In this process, the economic, political and social contexts will evolve, calling for a new round of guided evolution in terms of targets of selection, and range of variation around the targets. In short, the policy and governance frameworks are to be understood in terms of managing the process of variation, selection and replication in the Darwinist evolution of systems.

### 3.3. Combining Systems Public Affairs with market strategy

Table 1 summarizes SPA’s goals and how they relate to the new biology. First, strengthen the relevance of the non-market environment in the purpose of business through corporate law as well as regulation and public law. This is not something that businesses can do on their own. Second, promote problem-solving purposes through business innovations that identify ways of profitably solving problems of others. Third, relax external environment boundary conditions through cooperation and partnerships with other organizations and the public sector.

For these goals to contribute to enhanced corporate value in a new environment of intensified competition there must be a strategic combination of market and non-market strategies. Since the appropriate legislative, executive and judicial framework for business activity cannot simply be presupposed, the non-market strategies of business must contribute to the construction of such a framework whereby profit can be earned through problem solving rather than problem creation. A business can only achieve competitive edge in its industry and markets by combining its political and social activities through ‘market and non-market strategic alignment’ [65] with its business goals. Critical to this is the promotion of social and political capital, which together we describe as ‘non-market capital’ [65]. SPA activities by businesses go beyond legal compliance to incorporate social values in corporate purposes.

The requisite legal framework is one that reconciles the pursuit of social values with the fiduciary duty of directors to shareholders. Such a framework requires co-creation by business and government. Social capital is the ability to earn the trust of other parties and gain an advantageous position in the process. Information about the interests and problems of other parties with which businesses interact and cooperate is crucial to enhancing social capital [24]. Businesses need the resources, capabilities and internal delegated governance arrangements to collect, process and act on this information, thereby establishing a common purpose of promoting prosperity of both business and society.

Businesses participate in and support public policy decision-making processes not for profit as currently defined, but instead in pursuit of a legal and regulatory framework where profit-seeking activities of business derive from benefiting society and the environment. Critical to this is that political connections promote the private interests of businesses and their investors through enhancing not detracting from social capital [66].

Profit and value maximizing objectives have all too frequently resulted in businesses using their market domination, power and social influence for private and personal rather than social gain [67]. Business needs to see its purpose as being to enhance its interests and those of its investors through purposes of profiting from solving not creating problems for others. Likewise, governments should recognize where non-market environments do not permit business to adapt sufficiently rapidly by creating an environment for rules and regulations that promote a virtuous cycle of cooperation between business and society.

## 4. Systems Public Affairs cases

The arguments for top-down causation in biology are greatly strengthened by historical cases in which this approach has clearly been more successful in prediction of lower level properties than the other way round [31]. Case studies can perform the same role in management studies. This section looks at three cases to illustrate how businesses set socio-political goals in the context of non-market environment strategies. These cases come from South Korea, which is a country where there have been historically close relations between business and government. Capitalism came relatively late to Korea, and it had to develop in a nation steeped in Confucian ideas. Korean society serves as a test bed that shows the potential of non-market strategies.

These cases illustrate different levels of implementation of SPA strategies, but in all cases the role of government agencies was important. In Case A, it was important for the government to adopt a risk sharing agreement (RSA), as advocated by corporations and industry stakeholders, to control the price of new drugs while respecting the broader societal benefits including those of patients. Case B contributed to the economic development of rural communities as well as members of society, such as riders and part-time workers, by creating a structure that responded to social needs within the company through a combination of market and non-market strategies.

In Case C, the government and the National Assembly protected the interests of society in response to issues raised by relevant industry associations, small and medium-sized enterprises, and individual entrepreneurs. In fact, this was a world first in ensuring that large global platform companies could not charge excessive fees. These cases exemplify the deliberate establishment of partnerships between private and public institutions, underscoring the significant benefits that aligning private incentives with public incentives yield.

In the current non-market environment, company profit maximization often deviates in the short term from promoting the public interest. The case studies below demonstrate that a company can increase its shareholder value in an enlightened way by adopting a non-market, longer term strategy. Furthermore, the company serves the public interest even when this conflicts with shareholder value maximization in the short term. The cases therefore demonstrate the potential for non-market strategies and cooperation both to benefit society and make business more effective and resilient.

### 4.1. Case A: a global pharmaceutical company

Since 2008, all new medicines have been required to demonstrate efficacy and cost-effectiveness to be registered for National Healthcare Insurance coverage in Korea. These requirements were as challenging for global companies as Korean companies developing innovative new medicines, and very difficult for patients and their families seeking innovative new treatments.

A global pharmaceutical company wanted to launch a drug that both used new technologies and targeted a specific patient group. Demonstrating cost-effectiveness is problematic for such companies. Having received safety approval in 2009, the company started the registration process but faced a major issue in agreeing a price for the new drug with the government. The government asked for a 52% discount on what the company proposed, much below average prices of OECD member countries. Since drug prices in Korea are used as a reference price by other governments in Asia and the Middle East when determining their prices, agreeing a low price in Korea had wider international implications.

To counter the problem, the company decided to propose a new solution to the government that could harmonize corporate interests with societal benefits. The company proposed a risk sharing agreement (RSA) system to the Korean government that had already been implemented in Europe and other countries. The proposal was for the company and the government to share financial risks on National Health Insurance to satisfy needs of patients and doctors for the new medicine, while lessening the burden on health insurance finances. The company thought that the suggestion would benefit the public and society as well as the company and the entire pharmaceutical business.

It was not easy to introduce new policies. There were institutions with different positions within the Korean government, and some experts and NGOs expressed their opposition. Opponents believed that even if the financial risk was shared by pharmaceutical companies, it would adversely affect health insurance finances by driving up overall healthcare costs. Through the process of SPA activities, the company aimed to achieve a social agreement that satisfied the government, business and members of society. To enhance government and opponents’ understanding of the proposed solution, the company engaged with multi-level stakeholder groups, experts and related associations. The company also sought support of patients, doctors and associations, who were eagerly awaiting release of the new drug.

As a result, a pilot project was conducted by the government and the RSA system was adopted based on the results of the pilot to control the prices of new drugs as well as benefit patients. The company’s new medicine became the first in its class to be registered under the new policy and is now being used to prolong the lives of cancer patients for whom other treatments have failed or are not available.

By introducing the RSA system, the Korean government has been able to increase the effectiveness of treatment for patients with severe and rare diseases while expanding health insurance finances. Since the introduction of the system, most new medicines for severe and rare diseases have been introduced through this system, which not only helps the business of the company and its industry, but also provides opportunities for patients with severe and rare diseases to save lives through innovative new drugs.

Refunds paid by pharmaceutical companies through the RSA system are increasing every year, and according to the National Health Insurance Service, the funds increased from approximately USD 31.22 million in 2014 to approximately USD 271.15 million in 2022 and are estimated to reach around USD 446.15 million in 2025.

### 4.2. Case B: a global food company

A global food company entered the Korean market in the mid-1980s with a premium global brand. The company was doing business in Korea in line with global standards, so food safety and labour conditions were relatively good compared with local businesses. However, in the 2000s after the IMF crisis, the company faced growing difficulties regarding health, food safety, waste, recycling, labour and human rights in addition to patriotic sentiment around consumer, worker, NGO and political party interests. As a result, in 2016 the headquarters attempted to sell the Korean entity for USD 427.35 million but failed.

To accommodate the expanding influence of external stakeholders, the company decided to adopt non-market strategies and attempted to align its market strategy with a systems approach. While the company had previously utilized some non-market strategies, such as lobbying, the company’s management now recognized the need for a more comprehensive and systematic approach. The company set its business purpose as sustainable prosperity, consumer and environmental safety, and affordable, high-quality food.

To reverse existing negative sentiment, the company analysed its boundary conditions and interactions with other parties. Based on this, it established comprehensive SPA strategies. An external advisory group was formed to provide objective opinions on public perceptions of the business and promote cooperative relationships with relevant ministries of central and local government, workers, labour unions and NGOs.

In the process of implementing SPA, the company cooperated with the government by introducing advanced guidelines on food safety, and with local governments it helped develop the local economy by purchasing and advertising local agricultural products. The company also set an example for Korean society by raising working conditions for part-timers and riders above the Korean legal standard. In this way, the company took the lead in embracing social demands and offering alternative solutions to issues facing Korean society, such as providing opportunities to improve the treatment of vulnerable workers and taking the lead in hiring the elderly. Six years after the introduction of the SPA strategy, the company’s revenue doubled in 2021 at the same time as social value was enhanced for Korean society.

This case shows that a private company can contribute to economic development of rural communities as well as members of society, such as riders and part-time workers. It raised profits by creating a structure that responded to social needs within the company through a combination of market and non-market strategy.

### 4.3. Case C: a global platform company

In June 2020, a large global platform company that leads the global app market announced an extension of its in-app purchase (IAP) and 30% fee policy on game apps in Korea to all its apps, webtoons, music and video clips. This had the effect of preventing app developers and companies from using other payment systems. The company met with strong opposition from various parties including individual creators of webtoons and web novels, small-sized application businesses and start-ups, as well as global application developers. In early 2021, when a related bill (the IAP Act) was proposed in the Korean National Assembly, the US Embassy and the American Chamber of Commerce in Korea (AMCHAM), and big global platform companies expressed their strong opposition to it based on the Korea–US Free Trade Agreement.

A multi-level SPA strategic approach was taken by an application association to address and resolve concerns of key decision makers about trade issues and constitutional violations. A strategy was implemented of cooperating not just with direct business stakeholders, such as start-ups and global application business associations, but also with overseas organizations and governments affected by IAP Acts. Communication with the public was improved through forums, joint statements and published articles. As a result, concerns raised by the USA and the large global platform company were addressed, and the IAP Act, which eliminated the 30% fee, was passed in the National Assembly on 31 August 2021.

This was a world first in ensuring that large global platform companies could not charge excessive fees. The case illustrates that strategies implemented unilaterally without building social consensus can be very detrimental for business. After consultation, the large global platform company adopted strategies that contributed to the public interest and increased corporate value. Following passage of the law in Korea, the company announced in September 2022 that it would permit external payments to be made in India, Australia, Indonesia, Japan and Europe, and in April 2023 in the UK, with external payment systems other than its own.

Further case studies supporting the SPA approach can be found in Lee *et al.* [22].

## 5. Conclusion

This interdisciplinary study has revealed that recent developments in the fundamentals of biological science have major implications for the social sciences, as the purely reductionist models that once dominated biological science do so no more. On the contrary, the micro-level in biology has now been shown to have little predictive value [16], and instead, higher level models succeed in identifying what needs to exist at lower levels [31].

We have outlined the implications of this for economics and management studies, the most important of which are that social constraints need to be understood, anticipated and shaped by businesses and where that happens then evolutionary processes of business and society are accelerated relative to the natural selection processes of markets and regulation. That conclusion is reinforced by the clarifications of the principle of biological relativity we describe here. The interactions *between* levels of organization in biology are necessarily simultaneous since the boundary conditions of higher levels constrain lower levels *at all times*.

The implication for businesses is that they should incorporate SPA methods within the businesses themselves. It is better to anticipate non-market factors than to regard them as an afterthought. Active agency has driven evolution faster. We believe that can also happen in economics and business. The concept of Systems Public Affairs therefore responds to this need. Just as businesses manage their market strategies, so too non-market environments can be managed. This is critical for businesses to be able to pursue new opportunities and respond proactively and profitably to uncertainty. Analogous to how living organisms survive and thrive on cooperation in the presence of stochasticity, so too businesses and society prosper from cooperation for common prosperity in the face of radical uncertainty.

SPA ensures sustainable prosperity by viewing business and society through the lens of a systems approach to the non-market environment. It aligns private incentives with social and environmental interests by ensuring that companies profit from producing solutions not problems for others, thereby promoting the operation of markets, the profitability of companies, human flourishing and shared prosperity.

We are not the first to point to strong parallels between economics and living systems (e.g. [68]) but we believe we are the first to do so in the context of what Philip Ball [7] calls ‘The New Biology’.

## Data Availability

This article has no additional data.

## Footnotes

^1^
The influence of the biological analogy can be traced back to Alfred Marshall who stated that ‘the law of substitution – which is nothing more than a special and limited application of the law of survival of the fittest – tends to make one method of industrial organization supplant another when it offers a direct and immediate service at a lower price’ [21, p. 597].

^2^
The contrast between markets and non-market environments bears some resemblance to the conflict that Alfred Marshall faced in reconciling biological agents with physical markets [28,29].

^3^
As Sheila Dow notes, Alfred Marshall took a systems approach: ‘Marshall thus took a systems approach, at both the ontological and epistemological levels. He understood real processes in terms of systems, and also put forward his own system of thought. In this way he echoed Adam Smith’s evolutionary systems approach.’ [32, p. 8].

^4^
Metabolic networks can, and do, become multi-level in all living systems. In bacteria and in mitochondria, the functioning of metabolic networks ceases when the membrane is depolarized [50].
